# Functional limitation and happiness among older adults: the multiple mediating role of intergenerational support and intergenerational relationship

**DOI:** 10.3389/fpubh.2023.1249216

**Published:** 2023-10-12

**Authors:** Angdi Zhou, Yiwen Song, Xinru Li, Bingqin Hu, Yitong Chen, Peiyao Cui, Jinghua Li

**Affiliations:** ^1^School of Public Health, Jilin University, Changchun, Jilin Province, China; ^2^Fifth School of Clinical Medicine of Zhejiang Chinese Medical University (Huzhou Central Hospital), Huzhou, China

**Keywords:** older adults, happiness, intergenerational support, intergenerational relationship, instrumental activity of daily living

## Abstract

**Objectives:**

This study aimed to investigate the relationship between the functional limitation and happiness among Chinese older people and examined the multiple mediating effects of intergenerational support (instrumental support and financial support) and intergenerational relationship.

**Method:**

Data was drawn from the Chinese Family Panel Survey (CFPS) 2018 and 2020. Structural equation modeling was adopted to analyze the association among functional limitations, intergenerational support, intergenerational relationship, and the older adults happiness.

**Results:**

There was a significant association between the functional limitations and the lower happiness levels among the older adults. The instrumental support from adult children positively mediated the relationship between the functional limitation and the happiness. However, intergenerational relationships were reduced due to the dysfunction of the older adults, and played a negatively mediated role between the functional limitation and the happiness. In addition, instrumental and financial support play chain-mediating roles between functional limitation and happiness in older adults through intergenerational relationships.

**Conclusion:**

Intergenerational relationships and instrumental support enhance the happiness of older adults with functional impairments, but their role is limited by the changing structure of modern families. Long-term care programs combined with the intergenerational support from families for people with functional impairments in old age would be more effective to reduce the burden on adult children and maintain the quality of life of the older adults.

## 1. Introduction

With the rapid decline in fertility and the increase in life expectancy, population aging has become a global trend. However, with the prolongation of the life span of the older adults, functional impairment has become a major aging issue. It is estimated that the prevalence of functional limitation reached 14.3% over the age of 60 in China (1), and more than 20% in sixteen European countries (2).

Happiness, a kind of life evaluation, refers to peoples’ thoughts about the quality of their lives (3, 4). Researchers found aged people who perceived high happiness is related to longer lifespan (5, 6) and a reduced likelihood of all-cause mortality (7). However, with the decline of physical function, the happiness of the older adults perceived decreased (8). Inequalities in older people’s functional limitations are detrimental to their happiness and healthy aging. How to mitigate the negative impacts of functional limitation on the happiness of the older adults has come to the forefront.

Family is considered to be the most significant and sometimes even single source of support for older individuals in China and other Asian countries (9). Older adults usually adhere to traditional family-centered cultural values – the culture of filial piety. This intergenerational culture, refers to a family-centered cultural value that shapes individuals’ perceptions of intergenerational support and emphasizes the adult children’s obligations and caregiving responsibilities to their aging parents (10). Previous research has found that the high levels of emotional closeness and strong supports within the family contribute to healthy aging in older adults (10, 11). The increasing communication between two generations enabled the older adults to find meaningful ways to live and promote the elder’s happiness (12), while the lack of support of financial resources, housekeeping, or intimate emotions from the children may lead to the depressed mood and loneliness among older adults (13). However, these studies have paid less attention to the physical function of older adults. There are few reports on the suitability of the traditional Chinese model of aging to safeguard the happiness of those with functional impairments. The role of the adult children’s intergenerational support and intergenerational relationships in the relationship between functional limitations and the happiness of older adults deserves in-depth study.

### 1.1. Functional limitations, intergenerational support, and happiness

The instrumental and the financial supports are the main forms of the intergenerational support, and each plays a unique role in the intergenerational communication between the two generations. Financial support is the financial transfer between two generations (14, 15). Previous studies have concluded that in Asian cultural contexts, financial support is the evidence that children maintain the respect for their older adults parents, enhancing the happiness of older adults (16). For those older people with functional limitation, receiving financial support from adult kids will be useful to overcome the financial challenges and boost happiness, as their medical expenditure and daily care expense are more likely to be high due to their poor physical condition (17). Instrumental support is the assistance with household chores or other daily tasks (18). It is considered to be the most common form of support provided by the adult children, seen as a delayed intergenerational exchange to repay the parents for their early years (19). The declining physical function highlights the significance of the instrumental assistance for older individuals. Therefore, the intergenerational support, including the financial support and the instrument support may mediate the relationships between the functional limitation and the happiness among the older adults.

### 1.2. Functional limitations, intergenerational relationships, and happiness

Intergenerational relationship refers to an intangible structure of the companionship and the communication that reflects the closeness and trust between parent and child (20). Socioemotional selectivity theory (21) claims that the decline in independence in older adults’ lives at advanced ages has prompted a change in the way older adults interact socially, with people preferring to maintain stable relationships with emotionally close social partners and rejecting less meaningful acquaintances (22, 23), so they begin to seek the frequency of contact, material exchange, and intimate emotions that come with more solid social relationships (23), and more dependent on closely family members. Previous research found that a harmonious relationship with children may play an important supportive role for the older adults. A positive intergenerational relationship facilitates older adults with functional limitation to feel more care from their children, which is a dependent buffering factor between functional limitation and happiness (12, 20). Thus, the intergenerational relationship may be a key mechanism by which patterns affecting the happiness of the older adults.

### 1.3. Functional limitations, intergenerational support, intergenerational relationship, and happiness

Previous research found that the older generations who maintain frequent intergenerational contact with their families often report having a harmonious intergenerational relationship (24). If older persons consistently receive the intergenerational assistance from their offspring, this positive feedback loop will encourage older adults to describe their close ties with their children more favorably (25). Financial support can help relieve the stress of financial hardship, and instrumental support assists older adults with life tasks. These intergenerational exchanges increase the emotional connection between the two generations in their contact with the older adults by alleviating the loneliness brought on by a shrinking social circle (26). Therefore, from the perspective of older parents, receiving more financial resources and housekeeping assistance from their children is conducive to better intergenerational relationships and contributes to perceiving a higher level of happiness.

**Figure 1:**
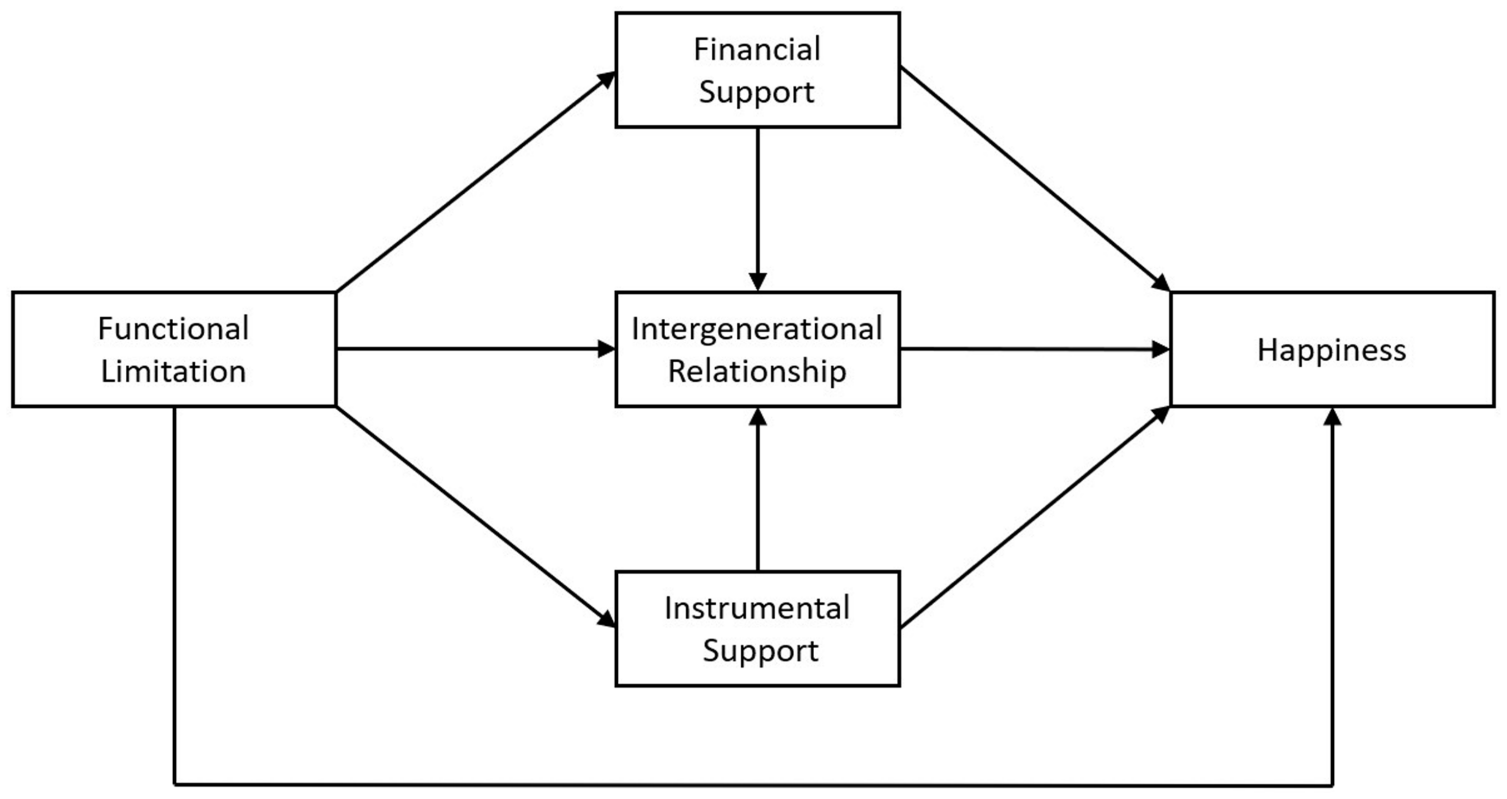
Conceptual framework.

Abundant studies discussed the predictors of the happiness in old aged people, few of them take all the above factors into consideration and a framework has yet to be established to explain the integrated relationship among these variables. Moreover, physical decline is a huge challenge that every older person has to face and threatens their quality of life. However, previous research has focused on the relationship between older people’s happiness and their family structure or social support (27), while neglecting older people’s physical functioning and specific support from close children. Whether the security provided by the children of older adults can buffer the adverse effects of functional limitations is the focus of our study. Thus, the present study aimed to investigate the relationship between the functional limitations and the happiness and examined whether and how this association is mediated by the intergenerational support and the intergenerational relationship. Research into this potential relationship will not only contribute to a comprehensive understanding of healthy aging in the older adults in a family context but will also provide the government and older adults health care social services with more information to improve the happiness of older people. The conceptual framework of the study is shown in Figure 1. The framework presents four main research hypotheses:

*Hypothesis 1*: Functional limitation in older people is negatively associated with happiness. Poor ability to perform daily activities in older people leads to lower happiness (H1).

*Hypothesis 2*: Intergenerational support mediates the relationship between functional limitations and happiness in older adults. Functional limitation in older people is positively associated with financial support (H2.1) and instrumental support (H2.2) from adult children, which can lead to higher levels of happiness.

*Hypothesis 3*: Intergenerational relationship mediates the relationship between functional limitations and happiness in older adults. Functional limitations in older people lead to higher levels of intergenerational relationship, which can lead to higher levels of happiness (H3).

*Hypothesis 4*: Intergenerational support and intergenerational relationship play a chain mediating effect between functional limitations and the happiness of the older adults. Financial (H4.1) and instrumental support (H4.2) will increase adult children’s intergenerational relationship with older adults.

## 2. Methods

### 2.1. Data source

The data is from two waves of China Family Panel Studies (CFPS) of 2018 and 2020. CFPS is a nationally representative, comprehensive social survey project that has been performed since 2010, organized by the Institute of Social Science Survey, Peking University. The studies were intended to collect individual, family, and community-level longitudinal data. CFPS contains detailed information such as demographic and educational outcomes, family dynamics, social relationships, migration, and health (28). CFPS was conducted in 25 provinces of China (excluding Hong Kong, Macau, Taiwan, Xinjiang, Tibet, Qinghai, Inner Mongolia, Ningxia, and Hainan). In this study, we used the adult questionnaire in CFPS, and our target respondents were adults aged 60 years old or above. We used data from CFPS 2020 and supplemented the sample that was surveyed only in CFPS 2018, ensuring that each sample had a unique ID and removing respondents without children. In China an increasingly large number of elder people tended to live on themselves. Even they did not live with their children they may still receive instrumental and financial support. Thus, respondents in this study included older adults people living with or without their children. To ensure the validity of the results, invalid data that answered “do not know” or refused to answer, were not applicable, or were left blank were removed from the subset containing 12,922 samples. After cleaning up the missing samples, the final sample content was 6,188.

### 2.2. Measures

#### 2.2.1. Functional limitation

The Instrumental Activities of Daily Living (IADL) is now commonly used in aging research to assess functional limitation in older people, which emphasize functional ability and independence in interacting with the environment (29). The current study used the IADL to assess functional limitation in older adults. The IADL questions in CFPS covered these seven tasks without assistance:(1) outdoor activities (walk about 300 meters, such as to the station, shopping center, parking lot); (2) eating (prepare a cup of tea or break the buns); (3) cooking (prepare meals for 1–2 people, wiping the table after the meal and washing dishes); (4) using public transportation (riding on public trams, busses, trains, and boats); (5) shopping (shopping in local stores and shopping centers, including shopping-related activities such as going in and out of the mall, selecting items, paying for them, and taking them home); (6) cleaning and hygiene; (7) laundry (including operating laundry equipment). Responses are dichotomous: 0 = be able to do so independently, 1 = unable to do so independently. The sum of the seven questions will be used to assess IADL difficulties, so higher scores indicate more severe IADL difficulties.

#### 2.2.2. Happiness

In CFPS, a single-item question was used to measure happiness: “Do you feel happy?” with an 11-point scale (0: very unhappy–10: very happy). Higher scores indicated high levels of happiness. Using a single question to measure happiness in older adults has been proven to be reliable by scholars (30–32).

#### 2.2.3. Intergenerational support

The majority of respondents in this study had more than one adult child (maximum 9), and respondents were asked about the intergenerational interactions between respondents and each of their children. In this study, intergenerational support was assessed along two dimensions: instrumental and financial support. Financial and instrumental support was asked through a combination of two questions, and if the first question was answered no, that support was recorded as zero. Respondents who answer yes will be asked next question about the specific frequency of support.

Instrumental support was measured by two questions (a): “Has your child (child’s name) helped you with household chores or taken care of you in the past 6 months? (b) “How often has your child (child’s name) helped you with household chores or cared for you in the past 6 months?” Responses were recorded using a five-point Likert scale ranging from 0 = never to 4 = almost every day.

Financial support was measured by two questions (a): “Please convert in-kind, did your child (child’s name) help you financially in the past 6 months? (b) “How much financial support have you received from your child (child’s name) in the past 6 months?”

Financial support and instrumental support will be measured using the sum of the support provided to the older adults by all adult children.

#### 2.2.4. Intergenerational relationship

The intergenerational relationship was measured by one question: “Are you close to your child (child’s name)? Answers to each item were assessed using a five-point Likert scale ranging from 1 = very poor to 5 = very close. We used the average score of intergenerational relationship for all adult children to indicate the level of intergenerational relationship.

#### 2.2.5. Control variables

Several demographic questions related to older adults were selected as control variables in this study, including age, gender (1 = male, 0 = female), marital status (1 = married, 0 = unmarried, including widowed, divorced, and unmarried), and self-rated income. Self-rated income levels were measured by a question: what level of income do you feel you have in your local area? Answers ranged from 1 (very low) - 5 (very high).

### 2.3. Data analysis

Data were analyzed using SPSS (IBM) version 24.0 and Amos 24. Structural Equation Modeling (path analysis) was adopted with Amos 24.0 to evaluate the hypothesized model (Figure 1). In the structural equation model, several indices were utilized in the evaluation of the hypothetical path and the modeling fitness. (1) For *χ*^2^, a smaller *χ*^2^ indicates the better model fit, and a non-significant (*p* > 0.05) *χ*^2^ shows that the model is suitable for the sample data (33). Nevertheless, if the sample size is too large, a well-fitted model normally produces a significant *χ*^2^ owing to the limitation of the likelihood ratio test (34) (2) For the comparative fit index (CFI) and Tucker and Lewis’ non-normed fit index (TLI), a model with values above 0.95 is considered a good fit, and a model with values above 0.90 is considered an adequate fit (33). (3) For the root mean square error of approximation (RMSEA), models with values less than 0.05 are considered a good fit (35).

## 3. Results

### 3.1. Descriptive findings

Table 1 presents the descriptive statistics of respondents. A total of 6,188 individuals aged 60 years or older (49.24% women) took part in the study. The middle age was 68.30, SD = 6.05. In terms of marital status, 15.42% of the older adults have no spouse, but every elder reported at least one child. A higher proportion of the older adults reported with a middle income (43.71%). The mean happiness score of the older adults respondents was 7.79 (range 0 ~ 10); The mean IDAL limitations was 0.51 (SD = 1.34, Range 0–7). According to intergenerational supports the older adults received, the mean scores of financial support and instrumental supports were 450 RMB (SD = 980) and 1.51 (SD = 2.20). The mean scores of intergenerational relationships were 4.31(SD = 0.70), with the range of 1–5.

**Table 1:** Descriptive statistics of variables: aged adults (aged 60+).

Variables	Frequency (*N*)	Percentage (%)	M ± SD
Gender			
Male	3,141	49.24	
Female	3,047	50.76	
Age	6,188		68.3 ± 6.05
Marital status			
Lived with a spouse	5,234	84.58	
Lived without a spouse	954	15.42	
Self-rated income			
Very low	599	9.68	
Low	913	14.75	
Middle	2,704	43.71	
High	986	15.93	
Very high	986	15.93	
IADL difficulty	6,188		0.51 ± 1.34
Financial support (thousands RMB per month) from child	6,188		0.45 ± 0.98
Instrumental support from child	6,188		1.51 ± 2.20
Intergenerational relationship	6,188		4.31 ± 0.70
Happiness	6,188		7.79 ± 2.09

### 3.2. Correlations among study variables

Pearson’s correlation coefficients are presented in Table 2. All measurements were significantly correlated with the happiness of the older adults; the highest correlation was for intergenerational relationship (*r* = 0.258, *p* < 0.001), and the lowest was for IADL difficulty (*r* = −0.040, *p* < 0.001).

**Table 2:** Pearson’s correlation coefficients.

Variables	1	2	3	4	5
1. IADL difficulty	1				
2. Fiancial support	0.026*	1			
3. Instrumental support	0.161***	0.074***	1		
4. intergenerational relationship	−0.036**	0.088***	0.076***	1	
5. Happiness	−0.040**	0.047***	0.083***	0.258***	1

*p < 0.05, **p < 0.01, ***p < 0.001.

### 3.3. Path analysis and model fit

The structural model fitted the data well. The chi-square value (*χ*^2^ = 98.886, *p* < 0.001, df = 13) was significant because of its sensitivity to a large sample size, but the results were satisfactory except for other goodness-of-fit indicators. CFI (0.925) was higher than 0.90 and RMSEA (0.038) was lower than 0.05. Results indicated that the model explained happiness in older adults with an explanation rate of 10.7%.

**Figure 2:**
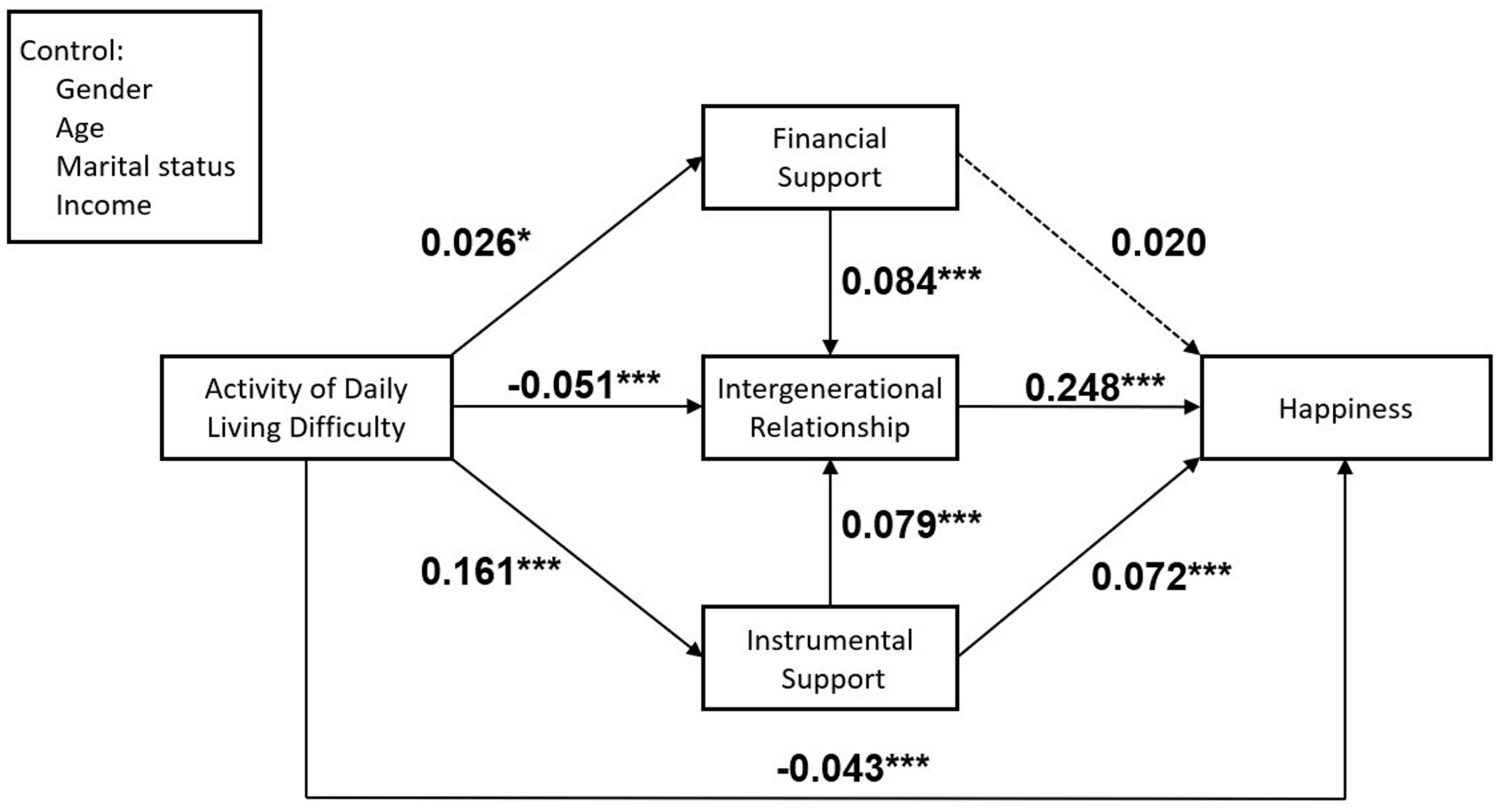
Standardized solutions for the structural model of family functional limitations, intergenerational support and happiness among the elderly.

The unstandardized and standardized path coefficients for the structural model are shown in Table 3, and the standardized solution for the structural model test is shown in Figure 2. For the sake of brevity, only paths for the main predictors are shown in this figure.

**Table 3:** Unstandardized and standardized path coefficients for the structural model.

Paths			*B*	*β*	S.E.	*p*
Instrumental support	<−--	IADL difficulty	0.266	0.161	0.021	<0.001
Financial support	<−--	IADL difficulty	0.019	0.026	0.009	0.039
Intergenerational relationship	<−--	IADL difficulty	−0.027	−0.051	0.007	<0.001
Intergenerational relationship	<−--	Financial support	0.060	0.084	0.009	<0.001
Intergenerational relationship	<−--	Instrumental support	0.025	0.079	0.004	<0.001
Happiness	<−--	Instrumental support	0.068	0.072	0.012	<0.001
Happiness	<−--	IADL difficulty	−0.066	−0.043	0.019	<0.001
Happiness	<−--	Intergenerational relationship	0.741	0.248	0.037	<0.001
Happiness	<−--	Financial support	0.043	0.020	0.026	0.102

### 3.4. Mediating effect test

Under the control of gender, age, marital status, and self-rated income, the Amos 24 was used to evaluate the 95% confidence interval of the mediating effect of functional limitation and happiness and the Bootstrap sample is 2000.

As shown in Figure 2 and Table 3, the higher the functional difficulty, the lower the level of happiness (*β* = −0.043, *p* < 0.001), which supports hypothesis 1. After including the mediating variables, functional limitation positively predicted instrumental support (*β* = 0.161, *p* < 0.001) and financial support (*β* = 0.026, *p* < 0.05). IADL difficulty was negatively correlated with intergenerational relationship (*β* = −0.051, *p* < 0.001), suggesting that difficulties with daily activities in older adults reduced the closeness of older adults to their children. Financial support is positively related to the intergenerational relationship (*β* = 0.084, *p* < 0.001); Instrumental support is positively related to the intergenerational relationship (*β* = 0.079, *p* < 0.001), and happiness of the older adults (*β* = 0.072, *p* < 0.001). Besides, the intergenerational relationship is positively related to happiness (*β* = 0.248, *p* < 0.001).

The multiple mediation effect was tested, and three significantly indirect paths are shown in Table 4: (1) the mediating effect of the path from functional limitation through instrumental support to happiness is 0.012, and the 95% confidence interval is (0.007, 0.016), excluding 0, supporting H2.2, (2) the mediating effect of the path from functional limitation through intergenerational relationship to happiness is −0.013, and the 95% confidence interval is (−0.019, −0.006), excluding 0, supporting hypothesis 3, (3) the chain mediating effect of the path from functional limitation through financial support and intergenerational relationship to happiness is 0.001, and 95% confidence interval excluded 0, supporting H4.1, and (4) the chain mediating effect of the path from functional limitation through instrumental support and intergenerational relationship to happiness is 0.003, and 95% confidence interval is (0.002, 0.004), excluding 0, supporting H4.2.

**Table 4:** Summary of the indirect effects.

Intermediate path	Standardized estimates	Standard error	95% confidence interval		Unstandardized estimates
			lower	upper	
IADLD→IS→HA	0.012	0.002	0.007	0.016	0.018
IADLD→IR→HA	−0.013	0.003	−0.019	−0.006	−0.020
IADLD→FS→IR→HA	0.001	<0.001	<0.001	0.001	0.001
IADLD→IS→IR→HA	0.003	0.001	0.002	0.004	0.005

IADLD, instrumental activity of daily living difficulty; FS, financial support; IS, instrumental support; IR, intergenerational relationship; HA, happiness.

## 4. Discussion

Using nationally representative data from the CFPS survey, this study tested the relationship between functional limitation and happiness among Chinese older adults. Findings suggested that functional limitation was negatively associated with happiness. The instrumental support and the intergenerational relationship each independently mediate this association, as well as instrumental and financial support also exert a chain mediating effect through intergenerational relationships. This study contributes to the existing literature on the happiness among older adults from a new inspect of function limitation. This study provides a new perspective on explaining the relationship between functional limitations and happiness in older adults by exploring the role of children in securing their happiness in later life.

Our finding indicated that ability to perform activities of daily living, as the basis for independent living and social activities for older people, is associated with the happiness among older people, which has been shown in previous literature (36, 37). Keeping a good physical function is a prerequisite and foundation for the happiness of the older adults. In the context of an increasingly aging population, preventing and slowing down the decline of physical function in older adults should be the key to responding to aging problem (38). Many studies have shown the implementation of comprehensive strategies such as moderate physical activity, good nutrition, chronic disease management, and good social security may benefit the healthy aging (39), hence relevant departments should pay more attention to the physical health issues of the older adults.

Our findings provide additional evidence for the crucial contributions that intergenerational relationships make to the happiness of older people. However, our study demonstrated that the functional limitations of older people hurt the intimate relationship between the adult children and their old parents. The physical dysfunction of the older adults increases the burden of care for adult children and actually damages intergenerational relationships. Under the context of Chinese filial piety culture, adult children have traditionally been the primary players in assisting vulnerable family members in their time of need (10). The increased care burden may lead to caregivers suffering adverse health outcomes such as loss of energy (40) and deterioration of interpersonal relationships (41), which subsequently increases intergenerational conflict and reduce intergenerational relationship quality (42). This embarrassing phenomenon also illustrates the dilemma of home care. As China’s aging deepens, the issue of older adults care cannot just rely on family member and other informal caregivers. The government should assume greater responsibility for increasing social care services for the older adults who are functionally impaired. Adult children and social care agencies should pay attention to the emotional needs of the older adults, especially family members should maintain communication with the older adults to maintain a harmonious family relationship.

Instrumental support from children positively mediates functional limitations and happiness. In addition, the chain mediators consisting of “instrumental support → intergenerational relationships” is also a way for functional limitations to affect the happiness of the older adults. Specifically receiving instrumental support does contribute to improving intergenerational relationship, and harmonious intergenerational relationship could increase the happiness of the older adults. In traditional Chinese cultural contexts, helping with housework do increase the contact between children and the older adults, which would improve the intergenerational relationship between children and the older adults (20, 43). However, with urbanization and socioeconomic development, and a decline in birth rate, more and more Chinese families are dominated by nuclear families, and the traditional three-generation family is becoming increasingly rare (44). The family model of aging is being challenged as fewer older adults people live with their children. It is difficult for children to give basic instrumental support to the older adults with functional disorders promptly (45). The government should pay attention to safeguarding the care needs of older adults people living alone and formulate relevant policies. Besides, contrary to our hypothesis, financial support from children is not associated with happiness among older adults. Although financial support acts as a chain mediator through intergenerational relationships, financial support from adult children does not play an important role in the happiness of older adults.

Overall, after considering the role of intergenerational support and intergenerational relationship, the effect of functional limitations on happiness in older adults remains negative. Our findings indicated that only depending on family support in ensuring the happiness of older adults with functional limitations is limited. As family structures change, patterns of care for older people change. Currently, many countries have established long-term care insurance services for the disabled older adults, and policymakers in China are devoted to developing a long-term care system that is affordable, wide coverage, and suited to the requirements of contemporary families (46). Past experience suggests that integrating social care services with family emotional support may be beneficial to happiness among older adults populations with functional impairment. Long-term care services should be a multilevel care system with individual, home, and community-based Services as the main body (47). The traditional Chinese culture of filial piety should also continue to play a role, and family members should give more emotional support to the older adults. Only through the joint efforts of individuals, families and society to strengthen the livelihood security of the older adults with disabilities can we better build a happy older adults society. Home and community-based services typically include daily living assistance such as bathing, feeding, and housework, as well as extensions of basic medical services such as rehabilitation guidance for chronic diseases. This will help the older adults with less functional limitations to maintain basic independence, autonomy and social participation (48). Nursing homes can help seniors who want professional medical advice and assistance to lead a normal life or accommodate the needs of seniors who demand a high quality of life in their later years in life.

## 5. Limitations

Some limitations should be acknowledged. First, it was a cross-sectional study and causality cannot be inferred. In future studies, longitudinal design studies should be adopted to test the causal relationships. Second, IADLs in CFPS was obtained through dichotomous questions. Low, medium, and high trivial variables may better respond to the IADL of older people. Third, there may be geographic and sociocultural differences in intergenerational relationships and financial support. However, the current study did not adequately consider these factors. Fourth, older adults may experience cognitive decline along with aging, and using purely subjective happiness data in the current study may led to biased results.

## 6. Conclusion

This study answers the question of how functional limitation affects the happiness of the older adults by constructing a chain mediation model. Specifically, Instrumental support positively mediates the relationship between functional limitations and happiness, whereas intergenerational relationships negatively mediate the relationship. In addition, instrumental support and intergenerational relationships also play positively and significantly chain mediated roles between functional limitations and happiness. We should be aware that, while family is important, it is not a panacea for the happiness of older adults in their later years. The study highlights the need to strengthen the social care system for older people to reduce the burden of family care.

## Data availability statement

Publicly available datasets were analyzed in this study. This data can be found at: http://www.isss.pku.edu.cn/cfps/wdzx/tcwj/index.htm.

## Ethics statement

The studies involving humans were approved by the biomedical ethics committee of Peking UniversityIRB00001052-14010. The studies were conducted in accordance with the local legislation and institutional requirements. The participants provided their written informed consent to participate in this study.

## Author contributions

AZ, XL, and JL: conceptualization. XL and YS: methodology and writing – review and editing. AZ and YS: software and data curation. JL: validation, resources, supervision, and funding acquisition. AZ, YS, BH, YC, and PC: formal analysis. XL, BH, YC, and PC: investigation. AZ: writing – original draft preparation. YS, BH, YC, and PC: visualization. XL and JL: project administration. All authors contributed to the article and approved the submitted version.
